# Mutation of Framework Residue H71 Results in Different Antibody Paratope States in Solution

**DOI:** 10.3389/fimmu.2021.630034

**Published:** 2021-03-02

**Authors:** Monica L. Fernández-Quintero, Katharina B. Kroell, Florian Hofer, Jakob R. Riccabona, Klaus R. Liedl

**Affiliations:** Department of General, Inorganic and Theoretical Chemistry, Center for Molecular Biosciences Innsbruck (CMBI), University of Innsbruck, Innsbruck, Austria

**Keywords:** antibodies, canonical clusters, molecular dynamics simulations, role of residue 71^H^, Markov-state modes

## Abstract

Characterizing and understanding the antibody binding interface have become a pre-requisite for rational antibody design and engineering. The antigen-binding site is formed by six hypervariable loops, known as the complementarity determining regions (CDRs) and by the relative interdomain orientation (V_H_–V_L_). Antibody CDR loops with a certain sequence have been thought to be limited to a single static canonical conformation determining their binding properties. However, it has been shown that antibodies exist as ensembles of multiple paratope states, which are defined by a characteristic combination of CDR loop conformations and interdomain orientations. In this study, we thermodynamically and kinetically characterize the prominent role of residue 71^H^ (Chothia nomenclature), which does not only codetermine the canonical conformation of the CDR-H2 loop but also results in changes in conformational diversity and population shifts of the CDR-H1 and CDR-H3 loop. As all CDR loop movements are correlated, conformational rearrangements of the heavy chain CDR loops also induce conformational changes in the CDR-L1, CDR-L2, and CDR-L3 loop. These overall conformational changes of the CDR loops also influence the interface angle distributions, consequentially leading to different paratope states in solution. Thus, the type of residue of 71^H^, either an alanine or an arginine, not only influences the CDR-H2 loop ensembles, but co-determines the paratope states in solution. Characterization of the functional consequences of mutations of residue 71^H^ on the paratope states and interface orientations has broad implications in the field of antibody engineering.

## Introduction

The rise of antibodies as important biotherapeutic proteins has sparked the interest in characterizing antibody structures and investigating structure–function relationships (1–3). Understanding the structural determinants and the involved conformational transitions governing antibody antigen recognition is critical for understanding antibody functions, in particular antibody specificity and consequently processes such as affinity maturation (4, 5). The antigen binding fragment (Fab) consists of a heavy and a light chain and can be divided into a constant and a variable domain. The variable fragment (Fv) exhibits the highest diversity of an antibody, as it is the focal point of somatic hypermutation and recombination events (6, 7). This high diversity of the Fv is concentrated on six hypervariable loops, also known as the complementarity determining regions (CDRs), which form the antigen binding site, the paratope. To facilitate the structure prediction of antibodies, five of these six CDR loops have been assigned to so-called canonical clusters, assuming that they can only adopt a limited number of backbone conformations (5, 8–11). Due to its unchallenged diversity in length, sequence and structure, no canonical clusters can be assigned for the CDR-H3 loop. Thus, structure prediction still remains challenging. In order to capture the high flexibility and diversity of the CDR-H3 loop and to functionally characterize all CDR loops, they have to be described as conformational ensemble in solution (12, 13). Within the obtained CDR loop ensembles in solution, also transitions between the majority of canonical clusters and additional dominant solution structures were observed.

Together with the CDR loops, the relative V_H_–V_L_ interdomain orientation plays an important role in determining the shape of the antigen binding site (4, 14–16). Various studies observed that mutations in the framework regions, in particular in the V_H_–V_L_ interface, can result in structural changes of the binding site and hence can influence antigen recognition. Additionally, allosteric effects during antibody antigen binding have also been reported, involving conformational rearrangements in the constant domains (C_H_1–C_L_) and the elbow angle (17–22).

The majority of V_H_–V_L_, C_H_1–C_L_ and elbow angle dynamics have been shown to occur in the low nanosecond timescale, while the slower components of the movements are strongly correlated with conformational changes in the CDR loops, which occur in the micro-to-millisecond timescale.

Based on these observations, antibodies were shown to exist as ensembles of paratope states in solution, which are defined by a characteristic combination of correlated CDR loop conformations and interdomain orientations. These paratope states interconvert into each other in the micro-to-millisecond timescale by synchronous loop and interdomain rearrangements.

**Figure 1:**
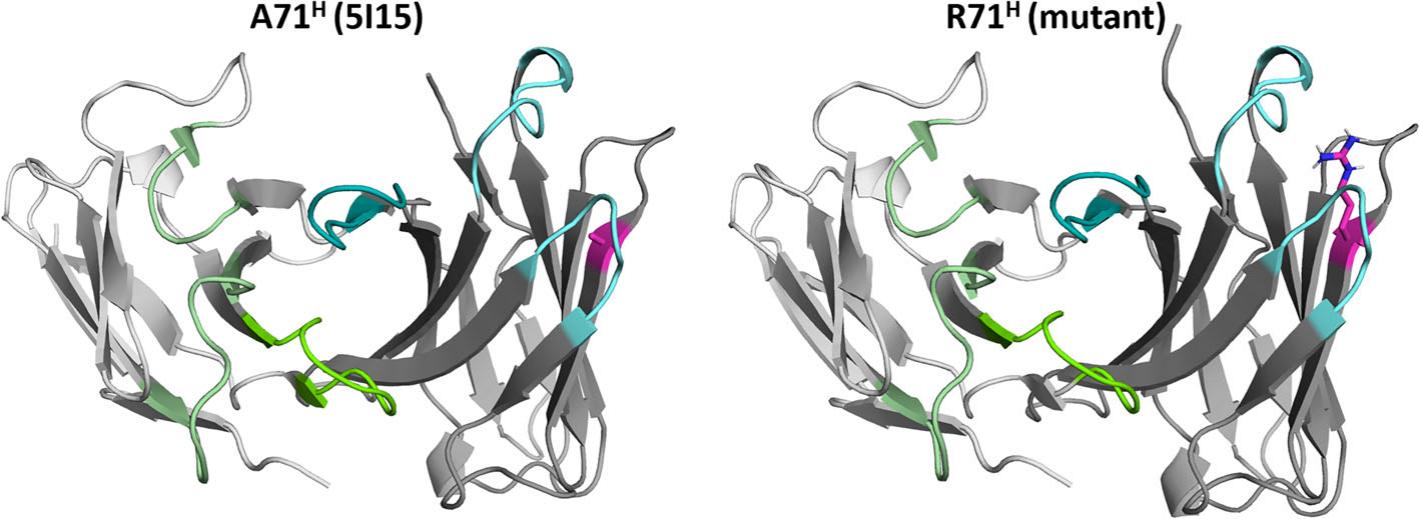
Structures of the germline IGHV1-69/IGKV1-39 antibody and the mutant (R71^H^) highlighting the position and the type of residue at position 71^H^. The residue at position 71^H^ is illustrated in magenta, while the heavy chain CDR loops are colored in cyan. The light chain CDR loops are depicted in green.

In this study we combine a well-established enhanced sampling technique with classical molecular dynamics simulations to kinetically characterize the influence of mutations of the prominent residue 71^H^ (Chothia nomenclature) (8, 23), on the conformational diversity on the CDR loops and the resulting paratope states in solution. **Figure 1** illustrates the position (HV4 loop) and residue type of 71^H^ with respect to the CDR loops, which are color-coded respectively.

## Methods

### Structure Preparation

As starting structure for the simulations, we used the human germline antibody IGHV1-69/IGKV1-39 with the PDB accession code 5I15 (24). The mutant starting structure for the simulations was prepared in MOE (Molecular Operating Environment, Chemical Computing Group, version 2020.01) by mutating residue 71^H^ to an arginine instead of an alanine. The mutated structure with the mutation at position 71^H^ will be further referred to as mutant. Additionally, the two structures were then also protonated using the Protonate3D tool (25, 26). Charge neutrality was ensured by utilizing the uniform background plasma approach in AMBER (27–29). Using the tleap tool of the AmberTools20 (27) package, the crystal structures were soaked in cubic water boxes of TIP3P water molecules with a minimum wall distance of 10 Å to the protein (30). The structures were described with the AMBER force field 14SB (31). The antibody fragments were carefully equilibrated using a multistep equilibration protocol (32).

### Metadynamics Simulations

To enhance the sampling of the conformational space, well-tempered bias-exchange metadynamics (33–35) simulations were performed in GROMACS (36, 37) with the PLUMED 2 implementation (38). As enhanced sampling technique, we chose metadynamics as it allows to focus the enhanced sampling on predefined collective variables (CV). The sampling is accelerated by a history-dependent bias potential, which is constructed in the space of the CVs (33, 35, 39). As collective variables, we used a well-established protocol, boosting a linear combination of sine and cosine of the ψ torsion angles of all CDR loops calculated with functions MATHEVAL and COMBINE implemented in PLUMED 2 (13, 38, 40–43). As discussed previously, the ψ torsion angle captures conformational transitions comprehensively (44). The underlying method presented in this paper has been validated in various studies against a large number of experimental results. The simulations were performed at 300 K in an NpT ensemble using the GPU implementation of the pmemd module (45) to be as close to the experimental conditions as possible and to obtain the correct density distributions of both protein and water. We used a Gaussian height of 10 kJ/mol. Gaussian deposition occurred every 1,000 steps and a biasfactor of 10 was used. 500 ns of bias-exchange metadynamics simulations were performed for the prepared Fab structures. The resulting trajectories were clustered with the program cpptraj (28, 46) using the average linkage hierarchical clustering algorithm with a distance cut-off criterion of 1.2 Å resulting in a large number of clusters. For the 5I15 antibody, we obtained 256 cluster representatives, while for mutant the clustering resulted in 279 cluster structures.The cluster representatives for the antibody fragments were equilibrated and simulated for 100 ns using the AMBER 20 (27) simulation package. Thus, the aggregated simulation time for the 5I15 Fab are 25.6 µs and for the mutant 27.9 µs. Additionally, in **Figure S2** the cluster representative for both antibody fragments is illustrated.

### Molecular Dynamics Simulations

As mentioned above, we performed for each obtained cluster representative 100 ns of classical molecular dynamics simulations. Molecular dynamics simulations were performed in an NpT ensemble using the pmemd.cuda module of AMBER 20 (28). Bonds involving hydrogen atoms were restrained with the SHAKE algorithm (47), allowing a time step of 2.0 fs. Atmospheric pressure (1 bar) of the system was set by weak coupling to an external bath using the Berendsen algorithm (48). The Langevin thermostat (49) was used to maintain the temperature during simulations at 300 K.

With the obtained trajectories, we performed a time-lagged independent component analysis (tICA) using the python library PyEMMA 2 employing a lag time of 10 ns. tICA was applied to identify the slowest movements of the investigated Fab fragments and consequently to obtain a kinetic discretization of the sampled conformational space (50).

tICA is a possible dimensionality reduction technique, detecting the slowest-relaxing degrees of freedom and facilitating the kinetic clustering, which is crucial for building an MSM (51). tICA is a linear transformation method, which linearly transforms a set of high-dimensional input coordinates to a set of output coordinates by finding a subspace of good reaction coordinates.

Based on the tICA conformational spaces, thermodynamics and kinetics were calculated with a Markov-state model (52) by using PyEMMA 2, which uses the k-means clustering algorithm (53) to define microstates and the PCCA+ clustering algorithm (54) to coarse grain the microstates to macrostates. Markov-state models are network models which provide valuable insights for conformational states and transition probabilities between them, as it is possible to accurately identify the boundaries between two states (52). The states are defined based on kinetic criteria, which allow identification of the boundaries between free energy wells. Basically, MSMs coarse-grain the system’s dynamics, which reflects the free energy surface and ultimately determines the system’s structure and dynamics. Thus, MSMs provide important insights and enhance the understanding of states and transition probabilities and facilitate a quantitative connection with experimental data (55, 56).

We performed tICA analyses and calculated Markov-state models of both investigated variants for the whole paratope and for all individual CDR loops.

The sampling efficiency and the reliability of the Markov-state model (*e.g*., defining optimal feature mappings) can be evaluated with the Chapman–Kolmogorov test (57, 58) by using the variational approach for Markov processes (59) and monitoring the fraction of states used, since the network states must be fully connected to calculate probabilities of transitions and the relative equilibrium probabilities. To build the Markov-state model we used the backbone torsions of the respective CDR loop, defined 150 microstates using the k-means clustering algorithm and applied a lag time of 10 ns.

The canonical cluster representatives for each CDR loop, extracted from the PyIgClassify database (60), were projected into the free energy surfaces of all individual CDR loops. We then used the respective macrostate ensembles to investigate correlations between the different paratope states and the relative V_H_ and V_L_ orientations.

### Relative V_H_ and V_L_ Orientations Using ABangle

ABangle is a computational tool (14, 15, 61, 62) to characterize the relative orientations between the antibody variable domains (V_H_ and V_L_) using six measurements (five angles and a distance). A plane is projected on each of the two variable domains. To define these planes, the first two components of a principal component analysis of 240 reference coordinates were used for V_H_ and V_L_ each. The reference coordinate set consists of C*α* coordinates of eight conserved residues for 30 cluster representatives from a sequence clustering of the non-redundant ABangle antibody data set. The planes were then fitted with those 240 coordinates, and consensus structures consisting of 35 structurally conserved C*α* positions were created for the V_H_ and V_L_ domain. Between these two planes, a distance vector C is defined. The six measures are then two tilt angles between each plane (HC1, HC2, LC1, LC2) and a torsion angle (HL) between the two planes along the distance vector C (dc). The ABangle script can calculate these measures for an arbitrary Fv region by aligning the consensus structures to the found core set positions and fitting the planes and distance vector from this alignment. This online available tool was combined with an in‐house python script to reduce computational effort and to visualize our simulation data over time. The in‐house script makes use of ANARCI (63) for fast local annotation of the Fv region and pytraj from the AmberTools package (27) for rapid trajectory processing.

## Results

**Figure 2:**
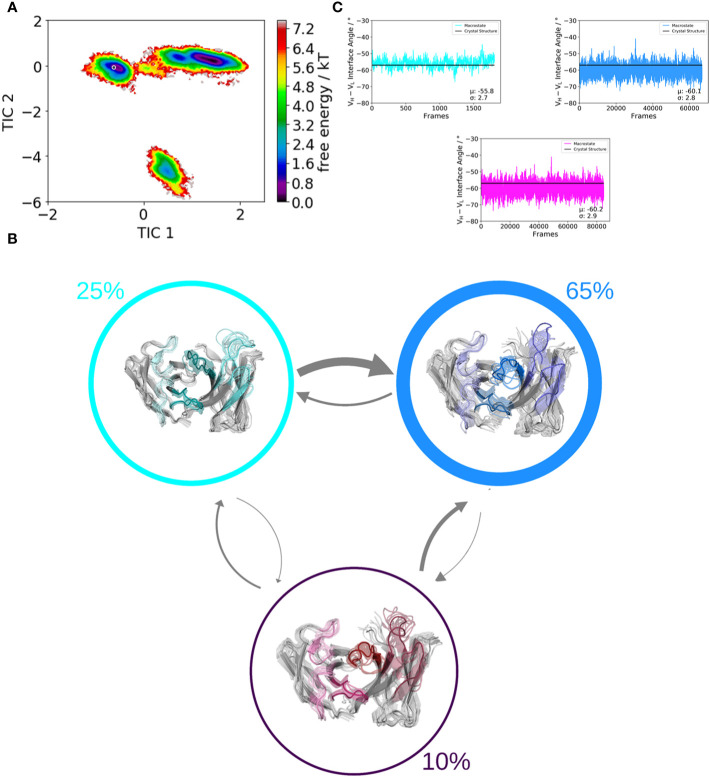
Paratope states in solution of the human germline IGHV1-69/IGKV1-39 antibody, characterized by the free energy surface, the respective Markov-state model and the relative V_H_–V_L_ interdomain orientations. Panel **(A)** shows the free energy surface of the whole paratope based on the backbone torsions of all CDR loops. Both the human germline IGHV1-69/IGKV1-39 antibody and the mutant (**Figure 3**) are projected into the same coordinate system. The gray dot represents the starting structure (PDB accession code: 5I15). Panel **(B)** illustrates the Markov-state model including the respective state probabilities. The transitions between different paratope states in solution occur in the micro-to-millisecond timescale and are represented by the thickness of the arrows. The macrostate arrangement corresponds to the free energy surface in panel **(A)**. Panel **(C)** depicts the relative V_H_–V_L_ interdomain orientations of the individual macrostates color-coded according to panel **(B)**.

**Figure 3:**
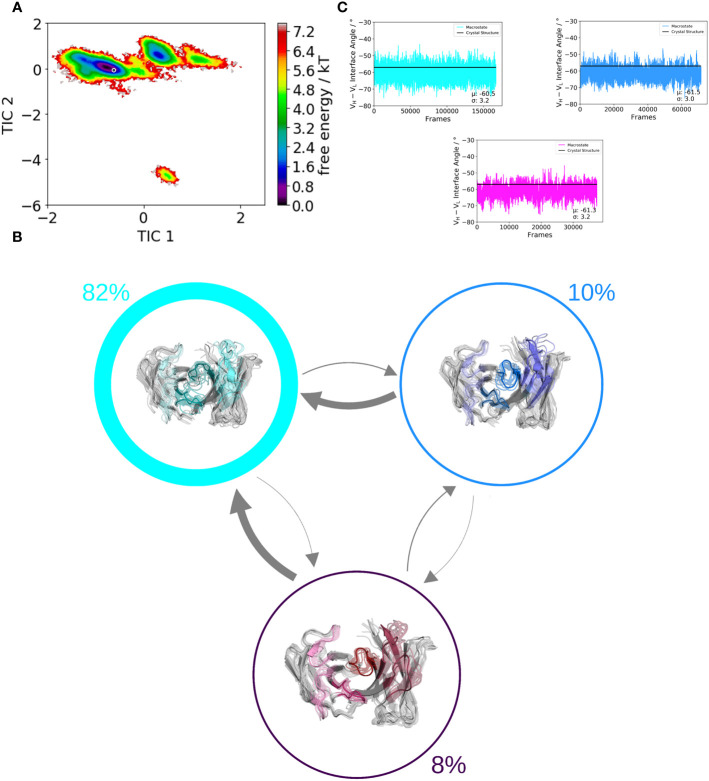
Paratope states in solution of the mutant antibody Fab, characterized by the free energy surface, the respective Markov-state model and the relative V_H_–V_L_ interdomain orientations. Panel **(A)** shows the free energy surface of the whole paratope based on the backbone torsions of all CDR loops. Both the human germline IGHV1-69/IGKV1-39 antibody (**Figure 2**) and the mutant are projected into the same coordinate system. The gray dot represents the starting structure (PDB accession code: 5I15). Panel **(B)** illustrates the Markov-state model including the respective state probabilities. The transitions between different paratope states in solution occur in the micro-to-millisecond timescale and are represented by the thickness of the arrows. The macrostate arrangement corresponds to the free energy surface in panel **(A)**. Panel **(C)** depicts the relative V_H_–V_L_ interdomain orientations of the individual macrostates color-coded according to panel **(B)**.

We use a well-established protocol combining enhanced sampling techniques and classical molecular dynamics to investigate the influence of residue 71^H^ on the whole paratope, the individual CDR loop dynamics and the respective relative V_H_–V_L_ orientations. We used the human germline IGHV1-69/IGKV1-39 antibody as starting structure for this study, which originally has an alanine on position 71^H^. We aim to kinetically and thermodynamically characterize the effect of mutating only alanine 71 to arginine on the resulting ensembles of paratope states. **Figures 2** and **3** show the free energy surfaces of the paratope of the IGHV1-69/IGKV1-39 antibody and the mutant in the same coordinate system, respectively. The calculated Markov-state model results for both investigated Fab variants in three macrostates, corresponding to the three paratope states in solution, which are illustrated in **Figure 2B** and **Figure 3B**. The most striking difference between the IGHV1-69/IGKV1-39 antibody and the mutant is the substantial population shift. The obtained macrostate trajectories from the Markov-state models were further used to calculate the relative interdomain orientations upon conformational changes in the paratope. For the IGHV1-69/IGKV1-39 antibody we clearly see a significant shift in the V_H_–V_L_ distribution, which is much smaller and not significant for the mutant. Thus, 71^H^ does not only strongly influence CDR loop dynamics, but also results in changes in the V_H_–V_L_ distributions.

To pinpoint the obtained global changes of the paratope to local CDR loop and interface rearrangements we also calculated free energy surfaces of the individual CDR loops.

### CDR-H1 Loop

**Figure 4:**
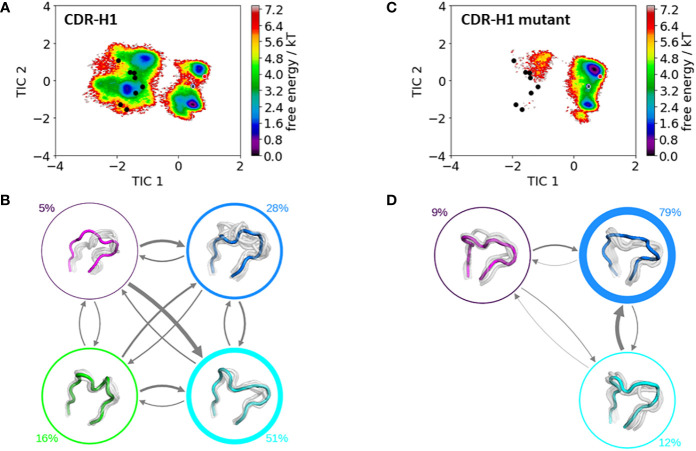
Comparison of the free energy surfaces and Markov-state models of the CDR-H1 loop of both the human germline IGHV1-69/IGKV1-39 antibody Fab and the mutant. Panel **(A)** illustrates the free energy surface of the CDR-H1 loop of the human germline antibody. The red dot represents the assigned canonical cluster structure (PDB accession code:1IC4), while the black dots represent all other available canonical cluster structures with a CDR-H1 loop length of 13 residues. The gray dot represents the starting structure (PDB accession code: 5I15). Panel **(B)** shows the corresponding macrostate ensembles with the respective state populations. The thickness of the circles reflects the state population. The thickness of the arrows corresponds to the transition timescales, which are in the micro-to-millisecond timescale. Panel **(C)** shows the Markov-state model of both the human germline and the mutant CDR-H1 loop, including the respective state probabilities. Again, the black dots represent all available canonical cluster structures of the CDR-H1 loop with a loop length of 13 residues, while the red dot shows the assigned canonical cluster structure (PDB accession code: 1IC4). The gray dot represents the starting structure. Panel **(D)** depicts the respective macrostate ensembles with respective state populations. The thickness of the circles reflects the state population. The thickness of the arrows corresponds to the transition timescales, which are in the micro-to-millisecond timescale.

**Figure 4** shows the free energy surfaces in the same coordinate system of the CDR-H1 loop of the IGHV1-69/IGKV1-39 antibody with and without the mutation of an alanine to an arginine at position 71^H^. Additionally, all available canonical clusters with the CDR-H1 loop length of 13 are projected into the free energy landscape colored in black, while the assigned canonical structure H1-13-4 (PDB accession code: 1IC4) is depicted in red. The gray dot represents the crystal structure of the IGHV1-69/IGKV1-39 antibody (PDB accession code: 5I15). What can immediately be noticed is the substantial rigidification of the conformational space, accompanied by a population shift, from the IGHV1-69/IGKV1-39 antibody (**Figure 4A**) to the mutant (**Figure 4C**). Furthermore, within the CDR-H1 loop conformational space of the IGHV1-69/IGKV1-39 antibody (**Figure 4A**) the majority of available canonical clusters are present within the sampled conformational ensemble; however, especially in this example, other dominant solution structures have to be considered, which are not apparent from X-ray structures. The free energy surface of the mutated antibody shown in **Figure 4C**, shows a substantial population shift towards the assigned canonical cluster and reveals a rigidification, which is also reflected in less sampled canonical clusters of the CDR-H1 loop. **Figures 4B**, **D** illustrate the resulting Markov-state models with the respective state probabilities. The thickness of the arrows corresponds to obtained transition times which occur in the micro-to-millisecond timescale. The type of residue of 71^H^ has already previously been shown to co-determine the canonical structure of the CDR-H2 loop (9).

### CDR-H2 Loop

**Figure 5:**
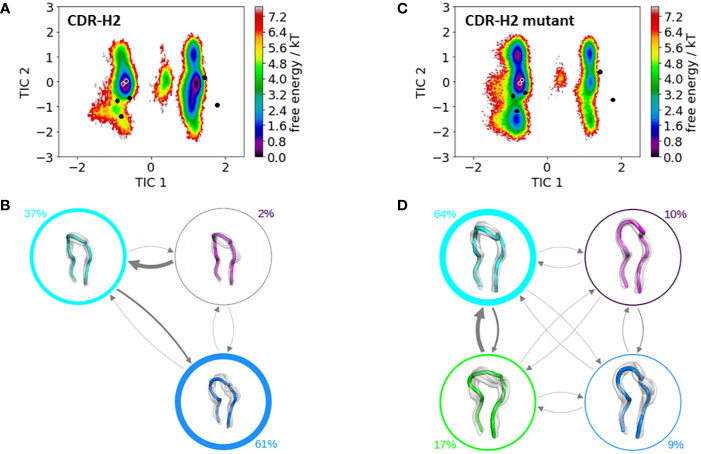
Comparison of the free energy surfaces and Markov-state models of the CDR-H2 loop of both the human germline IGHV1-69/IGKV1-39 antibody Fab and the mutant. Panel **(A)** illustrates the free energy surface of the CDR-H2 loop of the human germline antibody. The red dot represents the assigned canonical cluster structure (PDB accession code:2BDN), while the black dots represent all other available canonical cluster structures with a CDR-H2 loop length of 10 residues. The gray dot represents the starting structure (PDB accession code: 5I15). Panel **(B)** shows the corresponding macrostate ensembles with the respective state populations. The thickness of the circles reflects the state population. The thickness of the arrows corresponds to the transition timescales, which are in the micro-to-millisecond timescale. Panel **(C)** shows the Markov-state model of both the human germline and the mutant CDR-H2 loop, including the respective state probabilities. Again, the black dots represent all available canonical cluster structures of the CDR-H2 loop with a loop length of 10 residues, while the red dot shows the assigned canonical cluster structure (PDB accession code: 2BDN). The gray dot represents the starting structure. Panel **(D)** depicts the respective macrostate ensembles with respective state populations. The thickness of the circles reflects the state population. The thickness of the arrows corresponds to the transition timescales, which are in the micro-to-millisecond timescale.

**Figure 4** shows the resulting free energy surface and the Markov-state models including the respective state probabilities for the CDR-H2 loop with and without the mutation at position 71^H^. While conformational diversity of both CDR-H2 loop variants is comparable, mutating alanine at position 71^H^ to an arginine results in a strong population shift. Again, as described for the CDR-H1 also for the CDR-H2 loop, with a loop length of 10 residues, we sample the majority of available canonical clusters. Especially interesting is that the assigned canonical cluster H2-10-1 (PDB accession code: 2BDN), colored in red, for the IGHV1-69/IGKV1-39 antibody, lies in a local side-minimum, while the H2-10-2 (PDB accession code: 1SEQ) is close to the dominant minimum in solution (**Figure 5A**). **Figure 4C** shows the free energy surface of the mutant CDR-H2 loop, revealing this substantial population shift towards the assigned canonical conformation. The Markov-state model, including the state probabilities is depicted in **Figures 5B**, **D**. The IGHV1-69/IGKV1-39 antibody CDR-H2 loop results in three macrostates, while the mutant leads to four macrostates. The transition timescales between the macrostates lie again in the micro-to-millisecond timescale. As all CDR loops of the heavy chain are strongly correlated, also the influence of mutations at position 71^H^ on the observed CDR-H3 loop ensemble in solution is investigated.

### CDR-H3 Loop

**Figure 6:**
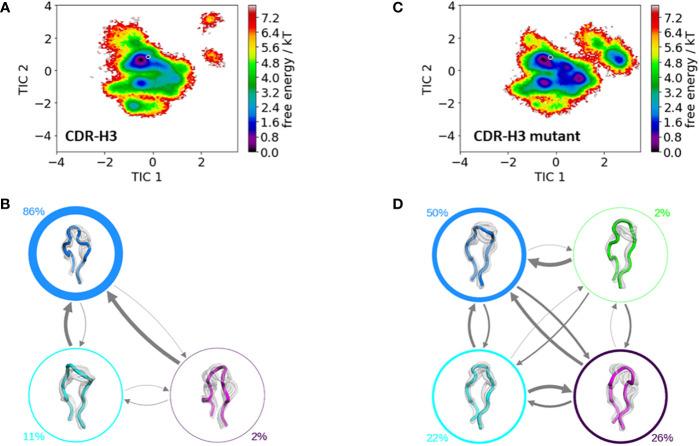
Comparison of the free energy surfaces and Markov-state models of the CDR-H3 loop of both the human germline IGHV1-69/IGKV1-39 antibody Fab and the mutant. Panel **(A)** illustrates the free energy surface of the CDR-H3 loop of the human germline antibody. The gray dot represents the starting structure (PDB accession code: 5I15). Panel **(B)** shows the corresponding macrostate ensembles with the respective state populations. The thickness of the circles reflects the state population. The thickness of the arrows corresponds to the transition timescales, which are in the micro-to-millisecond timescale. Panel **(C)** shows the Markov-state model of both the human germline and the mutant CDR-H3 loop, including the respective state probabilities. The gray dot represents the starting structure. Panel **(D)** depicts the respective macrostate ensembles with respective state populations. The thickness of the circles reflects the state population. The thickness of the arrows corresponds to the transition timescales, which are in the micro-to-millisecond timescale.

**Figure 6** shows the free energy surfaces of the CDR-H3 loop with and without mutating residue 71^H^ and reveals a higher conformational diversity for the mutant (**Figure 6C**). This higher flexibility is also reflected in the number of resulting macrostates, four macrostates for the mutant and three macrostates for the IGHV1-69/IGKV1-39 antibody CDR-H3 loop and is accompanied by population shifts. Thus, also the CDR-H3 loop ensemble in solution is strongly influenced by the type of residue of 71^H^.The Markov-state models for both variants are illustrated in **Figures 6B**, **D**, which show conformational rearrangements in microsecond timescale.

### Light Chain CDR Loops

These conformational rearrangements and population shifts in the heavy chain as a consequence of mutating residue 71^H^ from an alanine to an arginine can also be observed for the V_L_-CDR loops. Our results illustrated in **Figure S1** show that for all V_L_-CDR loops additional minima in solution can be identified. As all CDR loops are strongly correlated with each other, conformational changes observed for the heavy chain CDR loops have an effect on the light chain CDR loops as well. The flexibility of the CDR-H3 loop increases substantially as a consequence of the mutation and transfers this higher variability also on the V_L_-CDR loops.

## Discussion

In this study we thermodynamically and kinetically characterize the effect of a single point mutation at position 71^H^ for a human germline IGHV1-69/IGKV1-39 antibody on the paratope states in solution and give a structural and mechanistical explanation of the observed conformational changes. Various studies have already investigated the role of framework mutations on the CDR loops and the relative V_H_–V_L_ interdomain orientations based on X-ray structures (9, 64, 65). Even allosteric effects involving mutations in the C_H_1–C_L_ and the elbow angle have been reported to influence the antibody binding site and consequentially antibody affinity and specificity (17–22, 66–68). In particular, residue 71^H^, has been discussed to determine the canonical conformation of the CDR-H2 loop, according to whether there is a bulky residue or a small side-chain present and thus bringing the CDR-H1 and CDR-H2 loops closer to each other (16, 23, 69). Recently, it was also indicated that paratope states in solution, including the relative V_H_–V_L_ orientation, could be influenced by the type of residue of 71^H^ (68).

Previous studies described this prominent role of residue 71^H^ determining the CDR-H2 loop structure by considering crystal structures and sequences of naturally occurring antibodies and their respective variations. However, among these antibodies the CDR-H2 loop sequence not only differed, but they revealed also a high diversity in length, sequence, and structure of other CDR loops. Thus, in the course of antibody humanization, various studies focused on understanding the function of residue 71^H^ on structure, antigen-binding, and stability and engineered identical antibodies differing only in the type of residue at position 71^H^. Compared to the natural variations in this residue, functional differences could now be pinpointed to a single residue (69–72). Already from the earliest antibody engineering efforts, it has been observed that biases in the natural repertoire, which contribute to folding and stability, are selected and contribute successfully to the design of antibodies and synthetic libraries. Thus, also different residue types at position 71^H^ could be used to finetune the functions of antibodies and to balance the benefits of functional diversity by combining features of natural and engineered repertoires (73, 74).

Especially interesting is that the 71^H^ residue belongs to the Vernier-zone residues, which have been discussed to play a critical role in the humanization and for the rational design of antibodies in general as they can influence antibody specificity and affinity (65, 75, 76). Additionally, residue 71^H^ is part of the DE loop, also called H4 loop, which joins strands D and E on the heavy chain variable domain (9). The H4 loop has been traditionally considered to be part of the antibody framework; however, it has been shown not only for antibodies but also for T-cell receptors that the H4 loop can directly interact with the antigen and thus, influence antigen binding (9, 40). The fact that one single residue in the H4 loop can determine different paratope conformations in solution strongly supports the idea of highly correlated CDR loop movements, which interconvert into each other on the micro-to-millisecond timescale and favor specific interdomain orientations (12, 13, 41, 62, 68, 77, 78). Considering only one single static structure might not be sufficient to fully understand the consequences of point mutations on the resulting conformational diversity (42, 79). In line with these observations, we show for the human germline IGHV1-69/IGKV1-39 antibody strong population shifts towards different dominant paratope conformations in solution, when substituting 71^H^ from an alanine to an arginine (**Figure 2** and **Figure 3**). Additionally, we also identified shifts in the relative V_H_–V_L_ orientations depending on the type of residue of 71^H^.

To get a better understanding of the global changes, we also analyzed the influence of mutating residue 71^H^ on the individual CDR loops and their respective dynamics and were able to identify substantial differences in the obtained conformational ensembles in solution. For the CDR-H1 loop shown in **Figure 4**, we do not only see a strong population shift but also substantial rigidification when mutating residue 71^H^ to an arginine, which can be explained by strong hydrogen bond interactions of the arginine with the sidechains of a serine 245 (occurrence 20.24%), tyrosine 247 (occurrence 16.66%) and hydrogen bond and pi-stacking interactions with the backbone of phenylalanine 244 (occurrence 22.74 and 10.3%). **Figures 4A**, **C** illustrate the free energy surfaces of the CDR-H1 without and with the substitution, respectively. In line with previous studies (12, 13, 78), we observe that different canonical clusters lie within the same dominant minimum in solution, which is especially true for the germline IGHV1-69/IGKV1-39 antibody CDR-H1 loop. Thus, these canonical clusters might be combined. Another interesting aspect is that the slowest movement of the CDR-H1 loop, described by the TIC1, represents the conformational transition from the assigned canonical structure to all other available canonical structures with a CDR-H1 loop length of 13 residues.

Apart from sampling all available canonical structures, we are able to identify an additional solution structure, which represents the dominant minimum in solution and is not apparent from X-ray structures (**Figures 4A, B**). Upon substituting the alanine 71^H^ to an arginine, the populations of this dominant minimum are shifted towards the originally assigned canonical structure (**Figures 4C, D**).

The stabilization of the CDR-H1 as a consequence of the substitution of residue 71^H^ allows the CDR-H3 loop more degrees of freedom, which is reflected in the resulting conformational space illustrated in **Figure 6**. The increase in the flexibility of the CDR-H3 loop in **Figure 6C**, as a result of the stabilization of the CDR-H1 loop when mutating the alanine in position 71^H^ to an arginine, is also accompanied by a population shift (**Figures 6C, D**). For the CDR-H3 loop, due to its high diversity, no canonical structures were available and in agreement with previous studies, also here we see that the CDR-H3 loop needs to be characterized as conformational ensemble in solution (42, 78). As already discussed in literature, the CDR-H2 loop canonical conformation is strongly influenced by the bulkiness of the residue at position 71^H^, and even though we observe a similar conformational space, indeed strong population shifts towards different canonical structures of the obtained CDR-H2 loop ensembles in solution can be observed (**Figure 5**).

Similar to the observations for the CDR-H1 loop, also for the CDR-H2 loop the majority of canonical clusters are present within the sampled conformational ensemble in solution, clearly following the concept of conformational diversity. The concept of conformational diversity was proposed by Pauling and revived by Milstein and Foote, who demonstrated that the same antibody sequence can adopt various different conformations, which does not only influence their binding properties, but also increases the effective size of the antibody repertoire (80–82). Our results show that the individual CDR loops and the whole paratope, including the relative V_H_–V_L_ interdomain orientations, follow the concept of conformational diversity. **Figure S1** illustrates the free energy surfaces of the CDR-L1, CDR-L2, and CDR-L3 loops, to investigate if the mutation at position 71^H^ also influences the conformational diversity of the light chain CDR loops. In all light chain CDR loops, the free energy surfaces for the mutant reveal a broader conformational space similar to the increase in flexibility which was observed for the CDR-H3 loop. A potential explanation for this higher flexibility when substituting an alanine to an arginine could be that the introduction of arginine residues can enhance the promiscuity of antibodies (42, 83).

## Conclusion

In conclusion we observe in line with previous results that the type of residue at position 71^H^ does not only influence the neighboring CDR-H2 loop, but also induces conformational rearrangements in the whole paratope. Thus, mutating the prominent residue 71^H^ to either an alanine or an arginine results in different paratope states in solution, which also favor specific relative V_H_–V_L_ interdomain orientations. The results show that the antibody binding site exists as multiple paratope states in solution, with strongly correlated CDR loop and interdomain movements. This study raises the awareness of the strong correlations between the CDR loops and that one single static structure is not sufficient to capture the involved conformational changes and population shifts, which occur as a consequence of one single point mutation. Thus, we provide a new paradigm in the field of antibody engineering in the design of interconvertible paratope states in solution, which allows a full characterization of the antibody binding interface.

## Data Availability Statement

The original contributions presented in the study are included in the article/**Supplementary Material**. Further inquiries can be directed to the corresponding author.

## Author Contributions

All authors contributed to the article and approved the submitted version.

## Funding

This work was supported by the Austrian Science Fund (FWF) *via* the grant P30565 and P30737 and DOC 30.

## Conflict of Interest

The authors declare that the research was conducted in the absence of any commercial or financial relationships that could be construed as a potential conflict of interest.

## References

[1] Chiu ML Goulet DR Teplyakov A Gilliland GL. Antibody Structure and Function: The Basis for Engineering Therapeutics. Antibodies (Basel) (2019) 8:55. 10.3390/antib8040055 PMC6963682 31816964

[2] Kaplon H Reichert JM. Antibodies to watch in 2019. mAbs (2019) 11:219–38. 10.1080/19420862.2018.1556465 PMC6380461 30516432

[3] Kaplon H Muralidharan M Schneider Z Reichert JM. Antibodies to watch in 2020. mAbs (2020) 12:1703531. 10.1080/19420862.2019.1703531 31847708 PMC6973335

[4] Colman PM. Structure of Antibody-Antigen Complexes: Implications for Immune Recognition. In: Dixon FJ, editor. Advances in Immunology. Amsterdam: Academic Press (1988). p. 99–132. 10.1016/S0065-2776(08)60364-8 3055855

[5] Alzari PM Lascombe MB Poljak RJ. Three-Dimensional Structure of Antibodies. Annu Rev Immunol (1988) 6:555–80. 10.1146/annurev.iy.06.040188.003011 2454644

[6] French D Laskov R Scharff M. The role of somatic hypermutation in the generation of antibody diversity. Science (1989) 244:1152. 10.1126/science.2658060 2658060

[7] Akiba H Tsumoto K. Thermodynamics of antibody–antigen interaction revealed by mutation analysis of antibody variable regions. J Biochem (2015) 158:1–13. 10.1093/jb/mvv049 25956164

[8] Chothia C Lesk AM Tramontano A Levitt M Smith-Gill SJ Air G. Conformations of immunoglobulin hypervariable regions. Nature (1989) 342:877–83. 10.1038/342877a0 2687698

[9] Kelow SP Adolf-Bryfogle J Dunbrack RL. Hiding in plain sight: structure and sequence analysis reveals the importance of the antibody DE loop for antibody-antigen binding. (2020) mAbs 12:e1840005. 10.1101/2020.02.12.946350 PMC7671036 33180672

[10] Martin ACR Thornton JM. Structural Families in Loops of Homologous Proteins: Automatic Classification, Modelling and Application to Antibodies. J Mol Biol (1996) 263:800–15. 10.1006/jmbi.1996.0617 8947577

[11] Al-Lazikani B Lesk AM Chothia C. Standard conformations for the canonical structures of immunoglobulins1. J Mol Biol (1997) 273:927–48. 10.1006/jmbi.1997.1354 9367782

[12] Fernández-Quintero ML Heiss MC Pomarici ND Math BA Liedl KR. Antibody CDR loops as ensembles in solution vs. canonical clusters from X-ray structures. mAbs (2020) 12:1744328. 10.1080/19420862.2020.1744328 32264741 PMC7153821

[13] Fernández-Quintero ML Math BF Loeffler JR Liedl KR. Transitions of CDR-L3 Loop Canonical Cluster Conformations on the Micro-to-Millisecond Timescale. Front Immunol (2019) 10:2652. 10.3389/fimmu.2019.02652 31803187 PMC6877499

[14] Dunbar J Fuchs A Shi J Deane CM. ABangle: characterising the VH–VL orientation in antibodies. Protein Engineer Design Selection (2013) 26:611–20. 10.1093/protein/gzt020 23708320

[15] Bujotzek A Lipsmeier F Harris SF Benz J Kuglstatter A Georges G. VH-VL orientation prediction for antibody humanization candidate selection: A case study. mAbs (2016) 8:288–305. 10.1080/19420862.2015.1117720 26637054 PMC4966660

[16] Foote J Winter G. Antibody framework residues affecting the conformation of the hypervariable loops. J Mol Biol (1992) 224:487–99. 10.1016/0022-2836(92)91010-M 1560463

[17] Adachi M Kurihara Y Nojima H Takeda-Shitaka M Kamiya K Umeyama H. Interaction between the antigen and antibody is controlled by the constant domains: normal mode dynamics of the HEL-HyHEL-10 complex. Protein Sci (2003) 12:2125–31. 10.1110/ps.03100803 PMC2366927 14500870

[18] Pritsch O Hudry-Clergeon G Buckle M Petillot Y Bouvet JP Gagnon J. Can immunoglobulin C(H)1 constant region domain modulate antigen binding affinity of antibodies? J Clin Invest (1996) 98:2235–43. 10.1172/JCI119033 PMC507672 8941639

[19] Zhao J Nussinov R Ma B. Antigen binding allosterically promotes Fc receptor recognition. null (2019) 11:58–74. 10.1080/19420862.2018.1522178 PMC6343797 30212263

[20] Sotriffer CA Rode BM Varga JM Liedl KR. Elbow Flexibility and Ligand-Induced Domain Rearrangements in Antibody Fab NC6.8: Large Effects of a Small Hapten. Biophys J (2000) 79:614–28. 10.1016/S0006-3495(00)76320-X PMC1300962 10919996

[21] Sotriffer CA Liedl KR Linthicum DS Rode BM Varga JM. Ligand-induced domain movement in an antibody fab: molecular dynamics studies confirm the unique domain movement observed experimentally for fab NC6.8 upon complexation and reveal its segmental flexibility11Edited by I. Wilson. J Mol Biol (1998) 278:301–6. 10.1006/jmbi.1998.1684 9571052

[22] Röthlisberger D Honegger A Plückthun A. Domain Interactions in the Fab Fragment: A Comparative Evaluation of the Single-chain Fv and Fab Format Engineered with Variable Domains of Different Stability. J Mol Biol (2005) 347:773–89. 10.1016/j.jmb.2005.01.053 15769469

[23] Tramontano A Chothia C Lesk AM. Framework residue 71 is a major determinant of the position and conformation of the second hypervariable region in the VH domains of immunoglobulins. J Mol Biol (1990) 215:175–82. 10.1016/S0022-2836(05)80102-0 2118959

[24] Teplyakov A Obmolova G Malia TJ Luo J Muzammil S Sweet R. Structural diversity in a human antibody germline library. mAbs (2016) 8:1045–63. 10.1080/19420862.2016.1190060 PMC4968113 27210805

[25] Labute P. Protonate3D: Assignment of ionization states and hydrogen coordinates to macromolecular structures. Proteins (2009) 75:187–205. 10.1002/prot.22234 18814299 PMC3056144

[26] Molecular Operating Environment (MOE). 1010 Sherbrooke St. West, Suite #910, Montreal, QC, Canada,H3A 2R7 (2020).

[27] Case DA Belfon K Ben-Shalom IY Brozell SR Cerutti DS Cheatham TE II. AMBER 2020, University of California, San Francisco. AMBER 2020, University of California, San Francisco. (2020).

[28] Roe DR Cheatham TE. PTRAJ and CPPTRAJ: Software for Processing and Analysis of Molecular Dynamics Trajectory Data. J Chem Theory Comput (2013) 9:3084–95. 10.1021/ct400341p 26583988

[29] Hub JS de Groot BL Grubmüller H Groenhof G. Quantifying Artifacts in Ewald Simulations of Inhomogeneous Systems with a Net Charge. J Chem Theory Comput (2014) 10:381–90. 10.1021/ct400626b 26579917

[30] Jorgensen WL Chandrasekhar J Madura JD Impey RW Klein ML. Comparison of simple potential functions for simulating liquid water. J Chem Phys (1983) 79:926–35. 10.1063/1.445869

[31] Maier JA Martinez C Kasavajhala K Wickstrom L Hauser KE Simmerling C. ff14SB: Improving the accuracy of protein side chain and backbone parameters from ff99SB. J Chem Theory Comput (2015) 11:3696–713. 10.1021/acs.jctc.5b00255 PMC4821407 26574453

[32] Wallnoefer HG Liedl KR Fox T. A challenging system: Free energy prediction for factor Xa. J Comput Chem (2011) 32:1743–52. 10.1002/jcc.21758 21374633

[33] Barducci A Bussi G Parrinello M. Well-Tempered Metadynamics: A Smoothly Converging and Tunable Free-Energy Method. Phys Rev Lett (2008) 100:20603. 10.1103/PhysRevLett.100.020603 18232845

[34] Biswas M Lickert B Stock G. Metadynamics Enhanced Markov Modeling of Protein Dynamics. (2018). 10.1021/acs.jpcb.7b11800 29338243

[35] Barducci A Bonomi M Parrinello M. Metadynamics. WIREs Comput Mol Sci (2011) 1:826–43. 10.1002/wcms.31

[36] Abraham MJ Murtola T Schulz R Páll S Smith JC Hess B. GROMACS: High performance molecular simulations through multi-level parallelism from laptops to supercomputers. SoftwareX (2015) 1–2:19–25. 10.1016/j.softx.2015.06.001

[37] Pronk S Páll S Schulz R Larsson P Bjelkmar P Apostolov R. GROMACS 4.5: a high-throughput and highly parallel open source molecular simulation toolkit. Bioinformatics (2013) 29:845–54. 10.1093/bioinformatics/btt055 PMC3605599 23407358

[38] Tribello GA Bonomi M Branduardi D Camilloni C Bussi G. PLUMED 2: New feathers for an old bird. Comput Phys Commun (2014) 185:604–13. 10.1016/j.cpc.2013.09.018

[39] Ilott AJ Palucha S Hodgkinson P Wilson MR. Well-Tempered Metadynamics as a Tool for Characterizing Multi-Component, Crystalline Molecular Machines. J Phys Chem B (2013) 117:12286–95. 10.1021/jp4045995 24028495

[40] Fernández-Quintero ML Seidler CA Liedl KR. T-Cell Receptor Variable β Domains Rigidify During Affinity Maturation. Sci Rep (2020) 10:4472. 10.1038/s41598-020-61433-0 32161287 PMC7066139

[41] Fernández-Quintero ML Heiss MC Liedl KR. Antibody humanization—the Influence of the antibody framework on the CDR-H3 loop ensemble in solution. Protein Engineer Design Selection (2020) 32(9):411–22. 10.1093/protein/gzaa004 PMC7098879 32129452

[42] Fernández-Quintero ML Loeffler JR Kraml J Kahler U Kamenik AS Liedl KR. Characterizing the Diversity of the CDR-H3 Loop Conformational Ensembles in Relationship to Antibody Binding Properties. Front Immunol (2019) 9:3065. 10.3389/fimmu.2018.03065 30666252 PMC6330313

[43] Fernández-Quintero ML Pomarici ND Seidler CA Loeffler JR Liedl KR. T-cell receptor CDR3 loop conformations in solution shift the relative VH-VL domain distributions. Front Immunol (2020) 11:1–12. 10.3389/fimmu.2020.01440 32733478 PMC7360859

[44] Ramachandran GN Ramakrishnan C Sasisekharan V. Stereochemistry of polypeptide chain configurations. J Mol Biol (1963) 7:95–9. 10.1016/S0022-2836(63)80023-6 13990617

[45] Salomon-Ferrer R Götz AW Poole D Le Grand S Walker RC. Routine Microsecond Molecular Dynamics Simulations with AMBER on GPUs. 2. Explicit Solvent Particle Mesh Ewald. J Chem Theory Comput (2013) 9:3878–88. 10.1021/ct400314y 26592383

[46] Shao J Tanner SW Thompson N Cheatham TE. Clustering Molecular Dynamics Trajectories: 1. Characterizing the Performance of Different Clustering Algorithms. J Chem Theory Comput (2007) 3:2312–34. 10.1021/ct700119m 26636222

[47] Miyamoto S Kollman PA. Settle: An analytical version of the SHAKE and RATTLE algorithm for rigid water models. J Comput Chem (1992) 13:952–62. 10.1002/jcc.540130805

[48] Berendsen HJC Postma JPM van Gunsteren WF DiNola A Haak JR. Molecular dynamics with coupling to an external bath. J Chem Phys (1984) 81:3684–90. 10.1063/1.448118

[49] Adelman SA Doll JD. Generalized Langevin equation approach for atom/solid-surface scattering: General formulation for classical scattering off harmonic solids. J Chem Phys (1976) 64:2375–88. 10.1063/1.432526

[50] Scherer MK Trendelkamp-Schroer B Paul F Pérez-Hernández G Hoffmann M Plattner N. PyEMMA 2: A Software Package for Estimation, Validation, and Analysis of Markov Models. J Chem Theory Comput (2015) 11:5525–42. 10.1021/acs.jctc.5b00743 26574340

[51] Pérez-Hernández G Noé F. Hierarchical Time-Lagged Independent Component Analysis: Computing Slow Modes and Reaction Coordinates for Large Molecular Systems. J Chem Theory Comput (2016) 12:6118–29. 10.1021/acs.jctc.6b00738 27792332

[52] Chodera JD Noé F. Markov state models of biomolecular conformational dynamics. Curr Opin Struct Biol (2014) 25:135–44. 10.1016/j.sbi.2014.04.002 PMC4124001 24836551

[53] Likas A Vlassis N Verbeek J. The global k-means clustering algorithm. Pattern Recognition (2003) 36:451–61. 10.1016/S0031-3203(02)00060-2

[54] Röblitz S Weber M. Fuzzy spectral clustering by PCCA+: application to Markov state models and data classification. Adv Data Anal Classification (2013) 7:147–79. 10.1007/s11634-013-0134-6

[55] Bowman RG Pande V Noé F. An Introduction to Markov State Models and Their Application to Long Timescale Molecular Simulation. (2014). 10.1007/978-94-007-7606-7

[56] Chodera JD Noé F. Markov state models of biomolecular conformational dynamics. Curr Opin Struct Biol (2014) 25:135–44. 10.1016/j.sbi.2014.04.002 PMC4124001 24836551

[57] Karush J. On the Chapman-Kolmogorov Equation. Ann Math Statist (1961) 32:1333–7. 10.1214/aoms/1177704871

[58] Miroshin RN. Special solutions of the Chapman–Kolmogorov equation for multidimensional-state Markov processes with continuous time. Vestnik St Petersburg U: Mathematics (2016) 49:122–9. 10.3103/S1063454116020114

[59] Wu H Noé F. Variational approach for learning Markov processes from time series data. (2017).

[60] Adolf-Bryfogle J Xu Q North B Lehmann A Dunbrack RL. PyIgClassify: a database of antibody CDR structural classifications. Nucleic Acids Res (2015) 43:D432–8. 10.1093/nar/gku1106 PMC4383924 25392411

[61] Bujotzek A Dunbar J Lipsmeier F Schäfer W Antes I Deane CM. Prediction of VH–VL domain orientation for antibody variable domain modeling. Proteins: Structure Function Bioinf (2015) 83:681–95. 10.1002/prot.24756 25641019

[62] Fernández-Quintero ML Hoerschinger VJ Lamp LM Bujotzek A Georges G Liedl KR. VH-VL interdomain dynamics observed by computer simulations and NMR. Proteins: Structure Function Bioinf (2020). 10.1002/prot.25872 PMC7317758 31904133

[63] Dunbar J Deane CM. ANARCI: antigen receptor numbering and receptor classification. Bioinf (Oxford England) (2016) 32:298–300. 10.1093/bioinformatics/btv552 PMC4708101 26424857

[64] Teplyakov A Obmolova G Malia TJ Luo J Muzammil S Sweet R. Structural diversity in a human antibody germline library. mAbs (2016) 8:1045–63. 10.1080/19420862.2016.1190060 PMC4968113 27210805

[65] Teplyakov A Obmolova G Malia TJ Raghunathan G Martinez C Fransson J. Structural insights into humanization of anti-tissue factor antibody 10H10. MAbs (2018) 10:269–77. 10.1080/19420862.2017.1412026 PMC5825201 29283291

[66] Adair JR Athwal DS Emtage JS. Humanised antibodies. (1999).

[67] Stanfield RL Zemla A Wilson IA Rupp B. Antibody Elbow Angles are Influenced by their Light Chain Class. J Mol Biol (2006) 357:1566–74. 10.1016/j.jmb.2006.01.023 16497332

[68] Fernández-Quintero ML Pomarici ND Math BA Kroell KB Waibl F Bujotzek A. Antibodies exhibit multiple paratope states influencing VH–VL domain orientations. Commun Biol (2020) 3:589. 10.1038/s42003-020-01319-z 33082531 PMC7576833

[69] Krauss J Arndt MAE Zhu Z Newton DL Vu BK Choudhry V. Impact of antibody framework residue VH-71 on the stability of a humanised anti-MUC1 scFv and derived immunoenzyme. Br J Cancer (2004) 90:1863–70. 10.1038/sj.bjc.6601759 PMC2409732 15150594

[70] Xiang J Sha Y Jia Z Prasad L Delbaere LTJ. Framework Residues 71 and 93 of the Chimeric B72.3 Antibody are Major Determinants of the Conformation of Heavy-chain Hypervariable Loops. J Mol Biol (1995) 253:385–90. 10.1006/jmbi.1995.0560 7473721

[71] Holmes MA Buss TN Foote J. Structural Effects of Framework Mutations on a Humanized Anti-Lysozyme Antibody. J Immunol (2001) 167:296. 10.4049/jimmunol.167.1.296 11418663

[72] Honegger A Malebranche AD Röthlisberger D Plückthun A. The influence of the framework core residues on the biophysical properties of immunoglobulin heavy chain variable domains. Protein Engineer Design Selection (2009) 22:121–34. 10.1093/protein/gzn077 19136675

[73] Rothe C Urlinger S Löhning C Prassler J Stark Y Jäger U. The Human Combinatorial Antibody Library HuCAL GOLD Combines Diversification of All Six CDRs According to the Natural Immune System with a Novel Display Method for Efficient Selection of High-Affinity Antibodies. J Mol Biol (2008) 376:1182–200. 10.1016/j.jmb.2007.12.018 18191144

[74] Hackel BJ Ackerman ME Howland SW Wittrup KD. Stability and CDR Composition Biases Enrich Binder Functionality Landscapes. J Mol Biol (2010) 401:84–96. 10.1016/j.jmb.2010.06.004 20540948 PMC3927142

[75] Makabe K Nakanishi T Tsumoto K Tanaka Y Kondo H Umetsu M. Thermodynamic Consequences of Mutations in Vernier Zone Residues of a Humanized Anti-human Epidermal Growth Factor Receptor Murine Antibody, 528. J Biol Chem (2008) 283:1156–66. 10.1074/jbc.M706190200 17947238

[76] Arslan M Karadag D Kalyoncu S. Conformational changes in a Vernier zone region: Implications for antibody dual specificity. Proteins: Structure Function Bioinf (2020) 88:1447–57. 10.1002/prot.25964 32526069

[77] Fernández-Quintero ML Kroell KK Heiss MC Loeffler JR Quoika PK Waibl F. Surprisingly fast interface and elbow angle dynamics of antigen-binding fragments. Front Mol Biosci (2020) 7:1–10. 10.3389/fmolb.2020.609088 33330636 PMC7732698

[78] Fernández-Quintero ML Kraml J Georges G Liedl KR. CDR-H3 loop ensemble in solution – conformational selection upon antibody binding. null (2019) 11:1077–88. 10.1080/19420862.2019.1618676 PMC6748594 31148507

[79] Fernández-Quintero ML Loeffler JR Bacher LM Waibl F Seidler CA Liedl KR. Local and Global Rigidification Upon Antibody Affinity Maturation. Front Mol Biosci (2020) 7:1–13 32850970 10.3389/fmolb.2020.00182 PMC7426445

[80] Wedemayer GJ Patten PA Wang LH Schultz PG Stevens RC. Structural Insights into the Evolution of an Antibody Combining Site. Science (1997) 276:1665. 10.1126/science.276.5319.1665 9180069

[81] Pauling L. A Theory of the Structure and Process of Formation of Antibodies*. J Am Chem Soc (1940) 62:2643–57. 10.1021/ja01867a018

[82] Foote J Milstein C. Conformational isomerism and the diversity of antibodies. PNAS (1994) 91:10370–4. 10.1073/pnas.91.22.10370 PMC45021 7937957

[83] Birtalan S Zhang Y Fellouse FA Shao L Schaefer G Sidhu SS. The Intrinsic Contributions of Tyrosine, Serine, Glycine and Arginine to the Affinity and Specificity of Antibodies. J Mol Biol (2008) 377:1518–28. 10.1016/j.jmb.2008.01.093 18336836

