# Supplementary material for: Mutation of Framework Residue H71 Results in Different Antibody Paratope States in Solution
Source: Front Immunol. 2021 Mar 2;12:630034. doi: 10.3389/fimmu.2021.630034 (PMC7960778; doi:10.3389/fimmu.2021.630034)
Supplement: Supplementary file 1 [file DataSheet_1.docx]

Supplementary Material


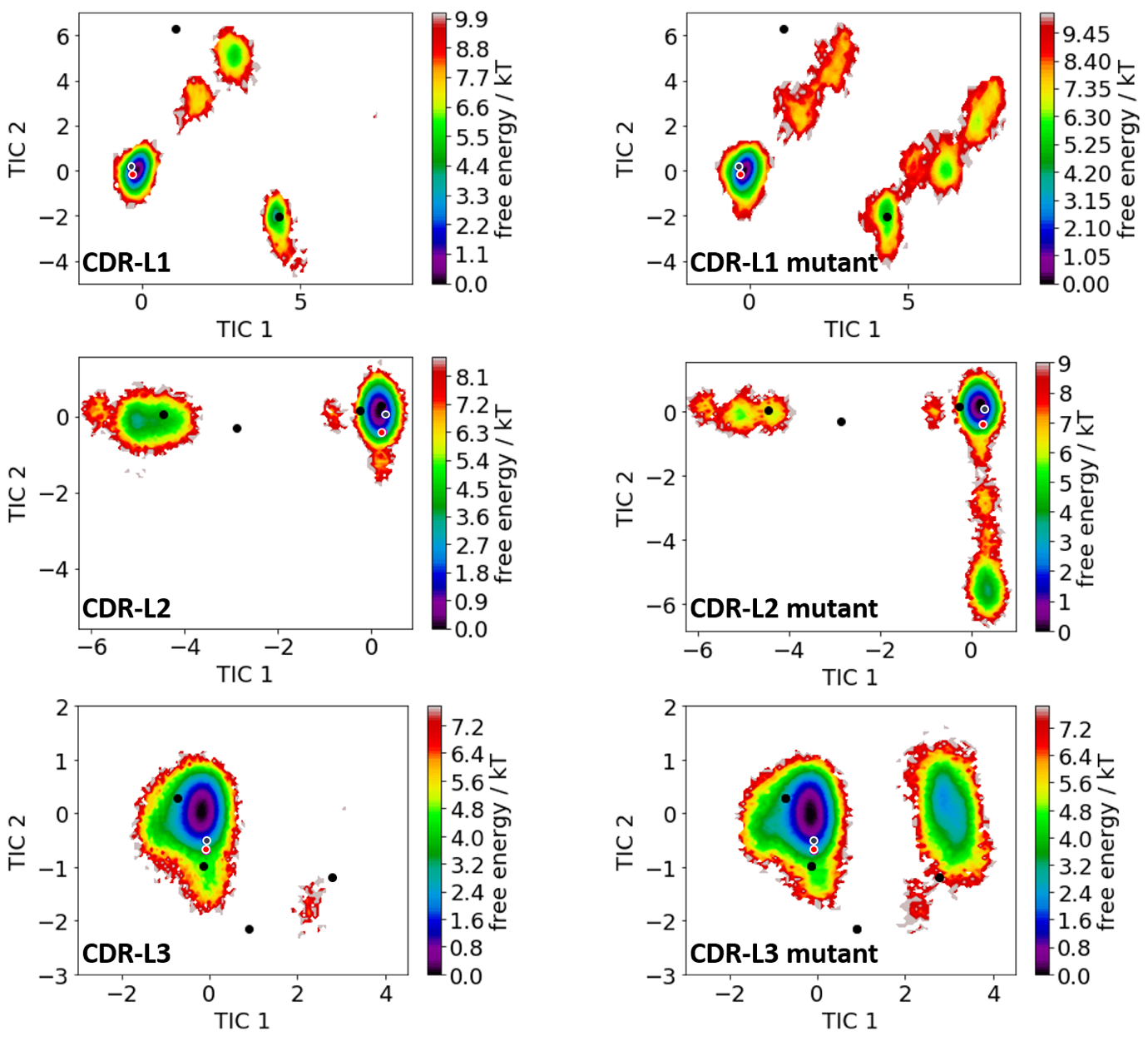


SI Figure S1: Free energy surfaces of the light chain CDR loops showing both the human germline antibody as well as the mutant Fab of the respective CDR loop in the same coordinate system. The black dots represent the available canonical clusters – CDR-L1 (L1-11*), CDR-L2 (L2-8*) and CDR-L3 (L3-9*), while the red dots show the assigned canonical cluster structure. The grey dot depicts the starting structure (PDB accession code: 5I15).


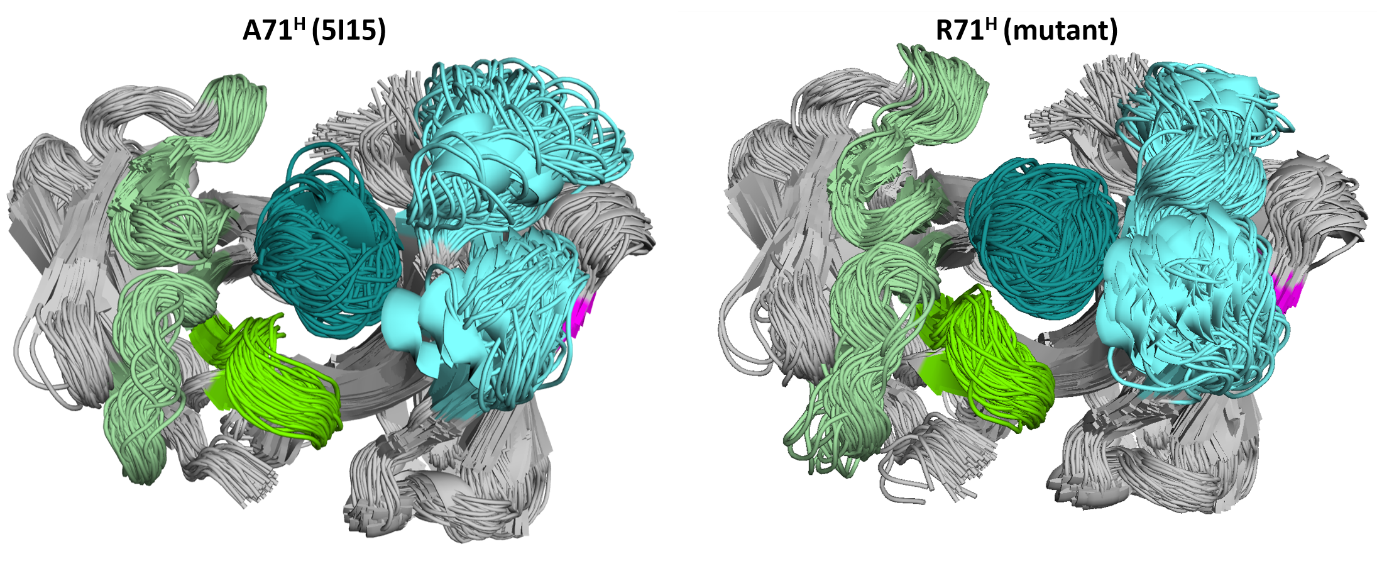


SI Figure S2: Ensembles of cluster representatives, which were used as starting structures for each 100 ns of molecular dynamics simulations for both the WT (5I15) as well as the mutant. For the WT we obtained 256 clusters, while for the clustering for the mutant resulted in 279 clusters.
